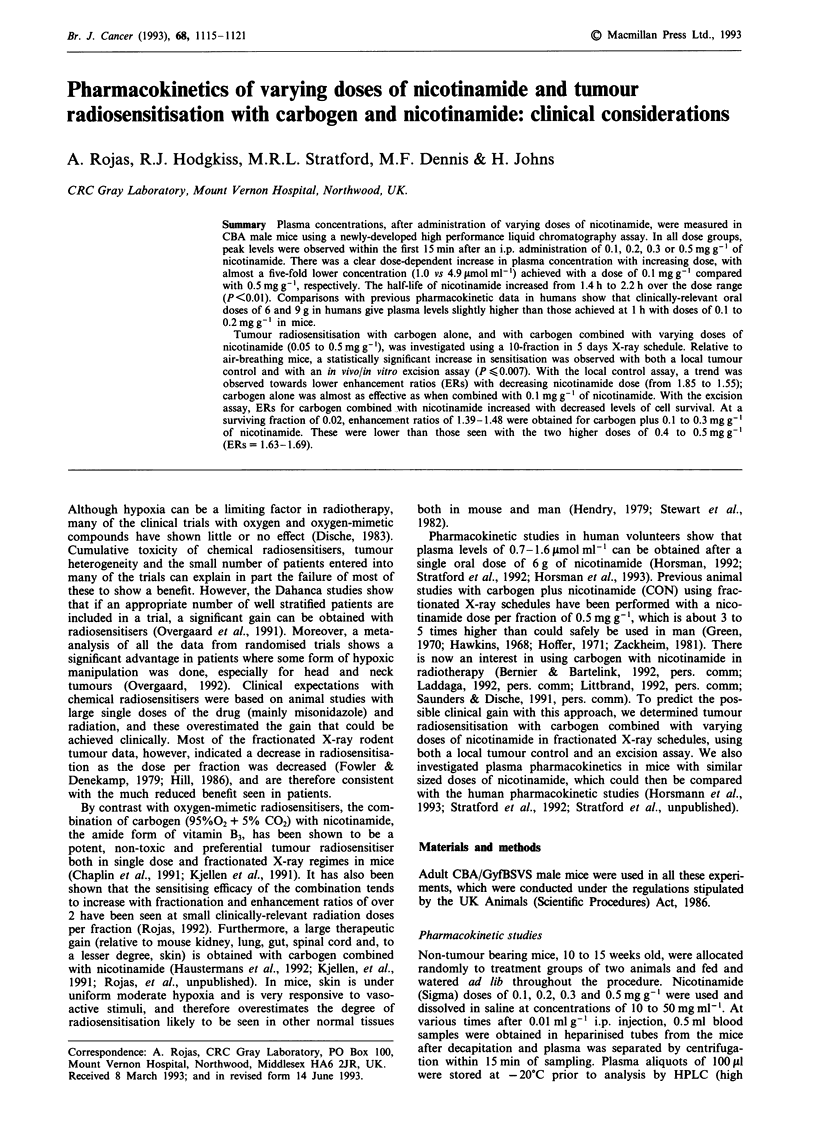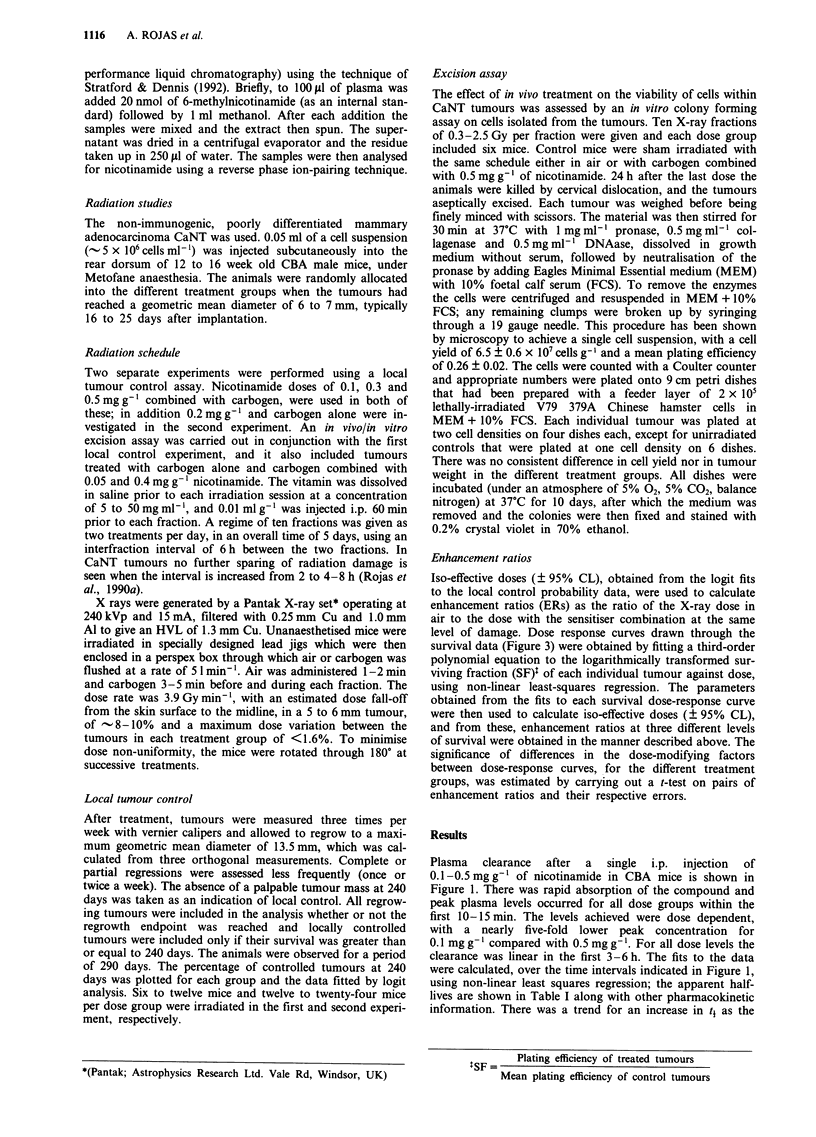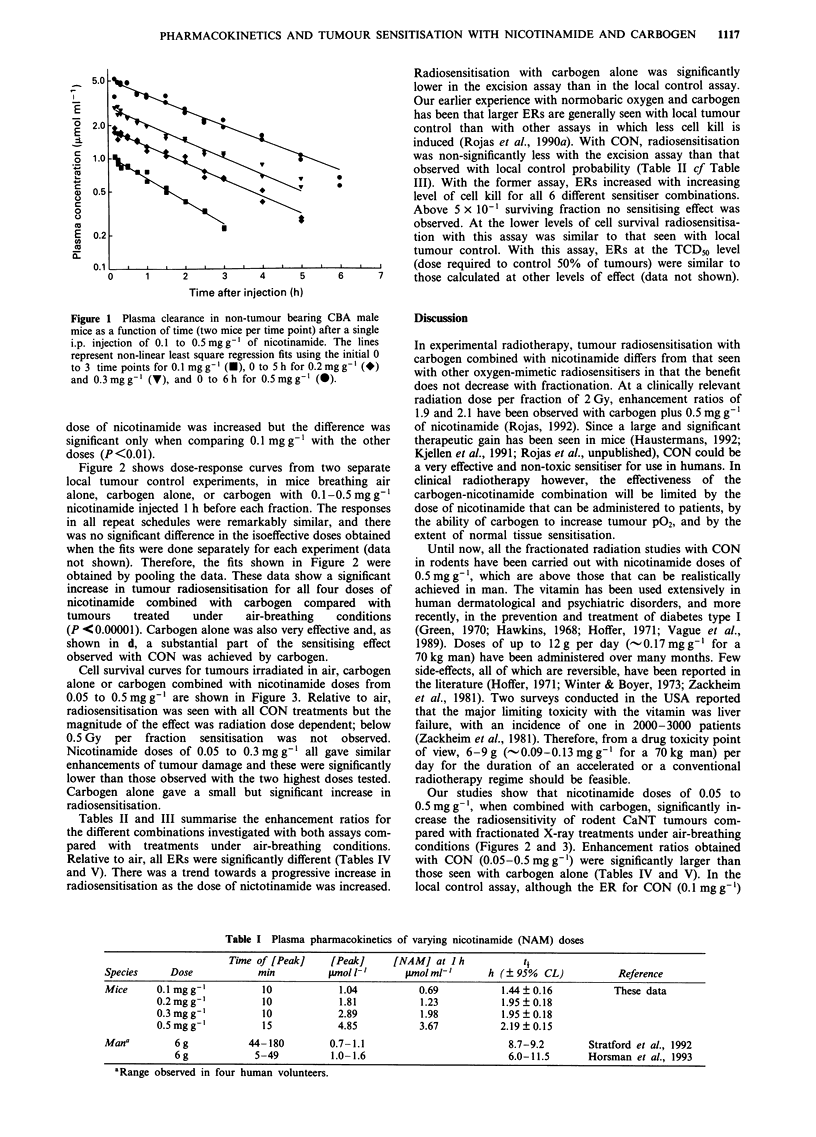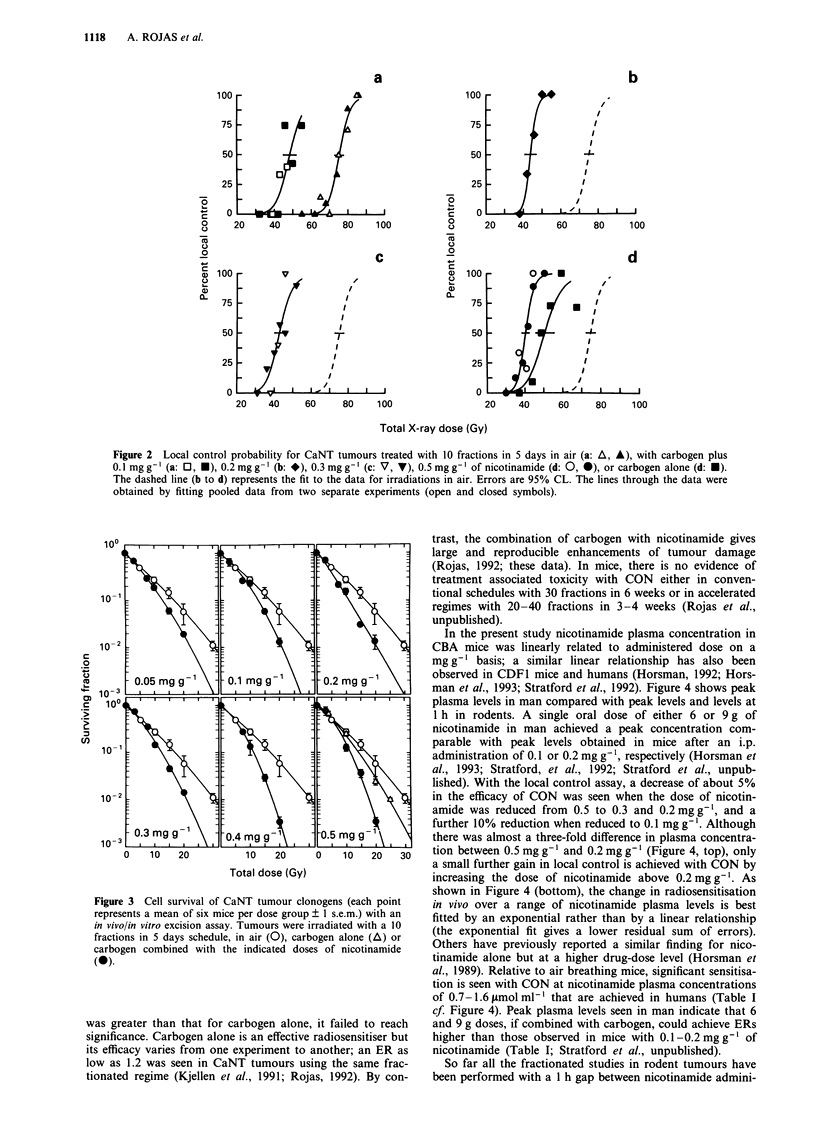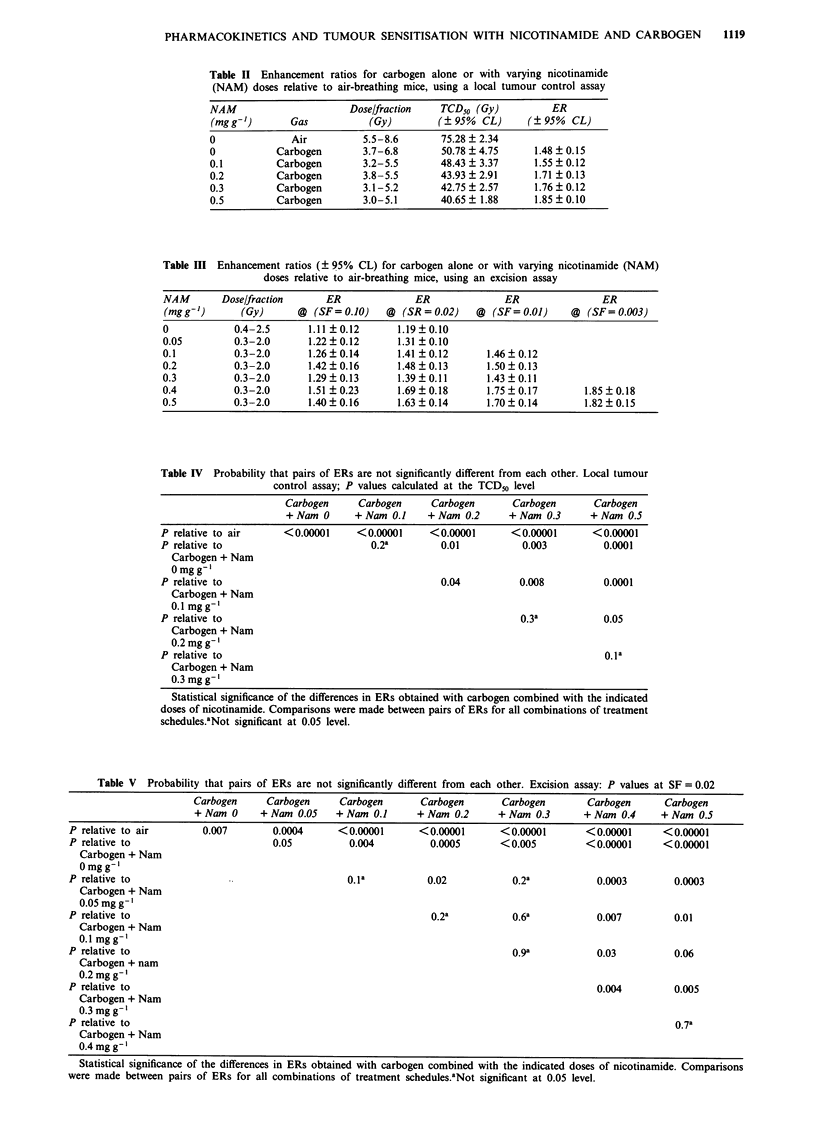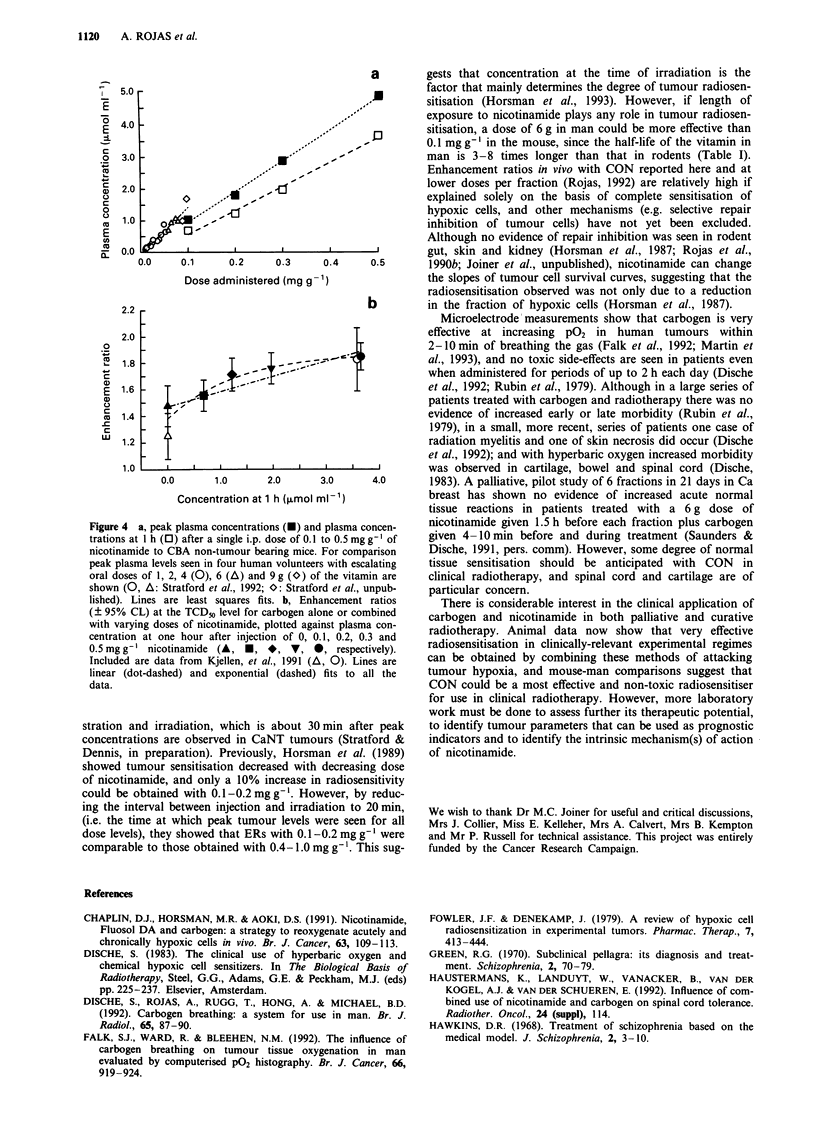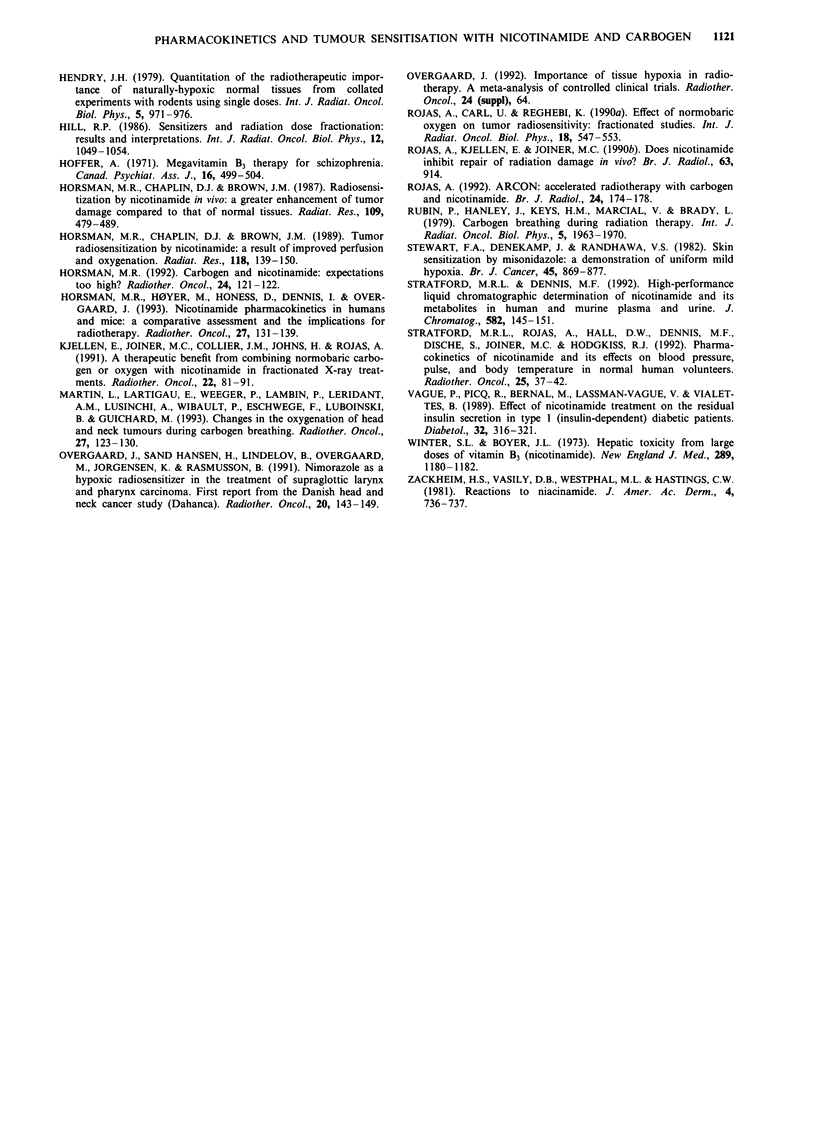# Pharmacokinetics of varying doses of nicotinamide and tumour radiosensitisation with carbogen and nicotinamide: clinical considerations.

**DOI:** 10.1038/bjc.1993.490

**Published:** 1993-12

**Authors:** A. Rojas, R. J. Hodgkiss, M. R. Stratford, M. F. Dennis, H. Johns

**Affiliations:** CRC Gray Laboratory, Mount Vernon Hospital, Northwood, UK.

## Abstract

Plasma concentrations, after administration of varying doses of nicotinamide, were measured in CBA male mice using a newly-developed high performance liquid chromatography assay. In all dose groups, peak levels were observed within the first 15 min after an i.p. administration of 0.1, 0.2, 0.3 or 0.5 mg g-1 of nicotinamide. There was a clear dose-dependent increase in plasma concentration with increasing dose, with almost a five-fold lower concentration (1.0 vs 4.9 mumol ml-1) achieved with a dose of 0.1 mg g-1 compared with 0.5 mg g-1, respectively. The half-life of nicotinamide increased from 1.4 h to 2.2 h over the dose range (P < 0.01). Comparisons with previous pharmacokinetic data in humans show that clinically-relevant oral doses of 6 and 9 g in humans give plasma levels slightly higher than those achieved at 1 h with doses of 0.1 to 0.2 mg g-1 in mice. Tumour radiosensitisation with carbogen alone, and with carbogen combined with varying doses of nicotinamide (0.05 to 0.5 mg g-1), was investigated using a 10-fraction in 5 days X-ray schedule. Relative to air-breathing mice, a statistically significant increase in sensitisation was observed with both a local tumour control and with an in vivo/in vitro excision assay (P < or = 0.007). With the local control assay, a trend was observed towards lower enhancement ratios (ERs) with decreasing nicotinamide dose (from 1.85 to 1.55); carbogen alone was almost as effective as when combined with 0.1 mg g-1 of nicotinamide. With the excision assay, ERs for carbogen combined with nicotinamide increased with decreased levels of cell survival. At a surviving fraction of 0.02, enhancement ratios of 1.39-1.48 were obtained for carbogen plus 0.1 to 0.3 mg g-1 of nicotinamide. These were lower than those seen with the two higher doses of 0.4 to 0.5 mg g-1 (ERs = 1.63-1.69).


*Br. J. Cancer* (1993), **68**, 1115–1121 © Macmillan Press Ltd., 1993

### Pharmacokinetics of varying doses of nicotinamide and tumour radiosensitisation with carbogen and nicotinamide: clinical considerations

A. Rojas, R.J. Hodgkiss, M.R.L. Stratford, M.F. Dennis & H. Johns

*CRC Gray Laboratory, Mount Vernon Hospital, Northwood, UK.*

**Summary** Plasma concentrations, after administration of varying doses of nicotinamide, were measured in CBA male mice using a newly-developed high performance liquid chromatography assay. In all dose groups, peak levels were observed within the first 15 min after an i.p. administration of 0.1, 0.2, 0.3 or 0.5 mg g$^{-1}$ of nicotinamide. There was a clear dose-dependent increase in plasma concentration with increasing dose, with almost a five-fold lower concentration (1.0 *vs* 4.9 μmol ml$^{-1}$) achieved with a dose of 0.1 mg g$^{-1}$ compared with 0.5 mg g$^{-1}$, respectively. The half-life of nicotinamide increased from 1.4 h to 2.2 h over the dose range ($P$<0.01). Comparisons with previous pharmacokinetic data in humans show that clinically-relevant oral doses of 6 and 9 g in humans give plasma levels slightly higher than those achieved at 1 h with doses of 0.1 to 0.2 mg g$^{-1}$ in mice.

Tumour radiosensitisation with carbogen alone, and with carbogen combined with varying doses of nicotinamide (0.05 to 0.5 mg g$^{-1}$), was investigated using a 10-fraction in 5 days X-ray schedule. Relative to air-breathing mice, a statistically significant increase in sensitisation was observed with both a local tumour control and with an *in vivo/in vitro* excision assay ($P \leqslant 0.007$). With the local control assay, a trend was observed towards lower enhancement ratios (ERs) with decreasing nicotinamide dose (from 1.85 to 1.55); carbogen alone was almost as effective as when combined with 0.1 mg g$^{-1}$ of nicotinamide. With the excision assay, ERs for carbogen combined with nicotinamide increased with decreased levels of cell survival. At a surviving fraction of 0.02, enhancement ratios of 1.39–1.48 were obtained for carbogen plus 0.1 to 0.3 mg g$^{-1}$ of nicotinamide. These were lower than those seen with the two higher doses of 0.4 to 0.5 mg g$^{-1}$ (ERs = 1.63–1.69).

Although hypoxia can be a limiting factor in radiotherapy, many of the clinical trials with oxygen and oxygen-mimetic compounds have shown little or no effect (Dische, 1983). Cumulative toxicity of chemical radiosensitisers, tumour heterogeneity and the small number of patients entered into many of the trials can explain in part the failure of most of these to show a benefit. However, the Dahanca studies show that if an appropriate number of well stratified patients are included in a trial, a significant gain can be obtained with radiosensitisers (Overgaard *et al.*, 1991). Moreover, a meta-analysis of all the data from randomised trials shows a significant advantage in patients where some form of hypoxic manipulation was done, especially for head and neck tumours (Overgaard, 1992). Clinical expectations with chemical radiosensitisers were based on animal studies with large single doses of the drug (mainly misonidazole) and radiation, and these overestimated the gain that could be achieved clinically. Most of the fractionated X-ray rodent tumour data, however, indicated a decrease in radiosensitisation as the dose per fraction was decreased (Fowler & Denekamp, 1979; Hill, 1986), and are therefore consistent with the much reduced benefit seen in patients.

By contrast with oxygen-mimetic radiosensitisers, the combination of carbogen (95%$O_2$ + 5% $CO_2$) with nicotinamide, the amide form of vitamin $B_3$, has been shown to be a potent, non-toxic and preferential tumour radiosensitiser both in single dose and fractionated X-ray regimes in mice (Chaplin *et al.*, 1991; Kjellen *et al.*, 1991). It has also been shown that the sensitising efficacy of the combination tends to increase with fractionation and enhancement ratios of over 2 have been seen at small clinically-relevant radiation doses per fraction (Rojas, 1992). Furthermore, a large therapeutic gain (relative to mouse kidney, lung, gut, spinal cord and, to a lesser degree, skin) is obtained with carbogen combined with nicotinamide (Haustermans *et al.*, 1992; Kjellen, *et al.*, 1991; Rojas, *et al.*, unpublished). In mice, skin is under uniform moderate hypoxia and is very responsive to vaso-active stimuli, and therefore overestimates the degree of radiosensitisation likely to be seen in other normal tissues both in mouse and man (Hendry, 1979; Stewart *et al.*, 1982).

Pharmacokinetic studies in human volunteers show that plasma levels of 0.7–1.6 μmol ml$^{-1}$ can be obtained after a single oral dose of 6 g of nicotinamide (Horsman, 1992; Stratford *et al.*, 1992; Horsman *et al.*, 1993). Previous animal studies with carbogen plus nicotinamide (CON) using fractionated X-ray schedules have been performed with a nicotinamide dose per fraction of 0.5 mg g$^{-1}$, which is about 3 to 5 times higher than could safely be used in man (Green, 1970; Hawkins, 1968; Hoffer, 1971; Zackheim, 1981). There is now an interest in using carbogen with nicotinamide in radiotherapy (Bernier & Bartelink, 1992, pers. comm; Laddaga, 1992, pers. comm; Littbrand, 1992, pers. comm; Saunders & Dische, 1991, pers. comm). To predict the possible clinical gain with this approach, we determined tumour radiosensitisation with carbogen combined with varying doses of nicotinamide in fractionated X-ray schedules, using both a local tumour control and an excision assay. We also investigated plasma pharmacokinetics in mice with similar sized doses of nicotinamide, which could then be compared with the human pharmacokinetic studies (Horsmann *et al.*, 1993; Stratford *et al.*, 1992; Stratford *et al.*, unpublished).

### Materials and methods

Adult CBA/GyfBSVS male mice were used in all these experiments, which were conducted under the regulations stipulated by the UK Animals (Scientific Procedures) Act, 1986.

### *Pharmacokinetic studies*

Non-tumour bearing mice, 10 to 15 weeks old, were allocated randomly to treatment groups of two animals and fed and watered *ad lib* throughout the procedure. Nicotinamide (Sigma) doses of 0.1, 0.2, 0.3 and 0.5 mg g$^{-1}$ were used and dissolved in saline at concentrations of 10 to 50 mg ml$^{-1}$. At various times after 0.01 ml g$^{-1}$ i.p. injection, 0.5 ml blood samples were obtained in heparinised tubes from the mice after decapitation and plasma was separated by centrifugation within 15 min of sampling. Plasma aliquots of 100 μl were stored at −20°C prior to analysis by HPLC (high

Correspondence: A. Rojas, CRC Gray Laboratory, PO Box 100, Mount Vernon Hospital, Northwood, Middlesex HA6 2JR, UK.
Received 8 March 1993; and in revised form 14 June 1993.

performance liquid chromatography) using the technique of Stratford & Dennis (1992). Briefly, to 100 µl of plasma was added 20 nmol of 6-methylnicotinamide (as an internal standard) followed by 1 ml methanol. After each addition the samples were mixed and the extract then spun. The supernatant was dried in a centrifugal evaporator and the residue taken up in 250 µl of water. The samples were then analysed for nicotinamide using a reverse phase ion-pairing technique.

*Radiation studies*

The non-immunogenic, poorly differentiated mammary adenocarcinoma CaNT was used. 0.05 ml of a cell suspension (~5 × $10^6$ cells $ml^{-1}$) was injected subcutaneously into the rear dorsum of 12 to 16 week old CBA male mice, under Metofane anaesthesia. The animals were randomly allocated into the different treatment groups when the tumours had reached a geometric mean diameter of 6 to 7 mm, typically 16 to 25 days after implantation.

*Radiation schedule*

Two separate experiments were performed using a local tumour control assay. Nicotinamide doses of 0.1, 0.3 and 0.5 mg $g^{-1}$ combined with carbogen, were used in both of these; in addition 0.2 mg $g^{-1}$ and carbogen alone were investigated in the second experiment. An *in vivo/in vitro* excision assay was carried out in conjunction with the first local control experiment, and it also included tumours treated with carbogen alone and carbogen combined with 0.05 and 0.4 mg $g^{-1}$ nicotinamide. The vitamin was dissolved in saline prior to each irradiation session at a concentration of 5 to 50 mg $ml^{-1}$, and 0.01 ml $g^{-1}$ was injected i.p. 60 min prior to each fraction. A regime of ten fractions was given as two treatments per day, in an overall time of 5 days, using an interfraction interval of 6 h between the two fractions. In CaNT tumours no further sparing of radiation damage is seen when the interval is increased from 2 to 4–8 h (Rojas *et al.*, 1990*a*).

X rays were generated by a Pantak X-ray set* operating at 240 kVp and 15 mA, filtered with 0.25 mm Cu and 1.0 mm Al to give an HVL of 1.3 mm Cu. Unanaesthetised mice were irradiated in specially designed lead jigs which were then enclosed in a perspex box through which air or carbogen was flushed at a rate of 5 l $min^{-1}$. Air was administered 1–2 min and carbogen 3–5 min before and during each fraction. The dose rate was 3.9 Gy $min^{-1}$, with an estimated dose fall-off from the skin surface to the midline, in a 5 to 6 mm tumour, of ~8–10% and a maximum dose variation between the tumours in each treatment group of <1.6%. To minimise dose non-uniformity, the mice were rotated through 180° at successive treatments.

*Local tumour control*

After treatment, tumours were measured three times per week with vernier calipers and allowed to regrow to a maximum geometric mean diameter of 13.5 mm, which was calculated from three orthogonal measurements. Complete or partial regressions were assessed less frequently (once or twice a week). The absence of a palpable tumour mass at 240 days was taken as an indication of local control. All regrowing tumours were included in the analysis whether or not the regrowth endpoint was reached and locally controlled tumours were included only if their survival was greater than or equal to 240 days. The animals were observed for a period of 290 days. The percentage of controlled tumours at 240 days was plotted for each group and the data fitted by logit analysis. Six to twelve mice and twelve to twenty-four mice per dose group were irradiated in the first and second experiment, respectively.

*Excision assay*

The effect of *in vivo* treatment on the viability of cells within CaNT tumours was assessed by an *in vitro* colony forming assay on cells isolated from the tumours. Ten X-ray fractions of 0.3–2.5 Gy per fraction were given and each dose group included six mice. Control mice were sham irradiated with the same schedule either in air or with carbogen combined with 0.5 mg $g^{-1}$ of nicotinamide. 24 h after the last dose the animals were killed by cervical dislocation, and the tumours aseptically excised. Each tumour was weighed before being finely minced with scissors. The material was then stirred for 30 min at 37°C with 1 mg $ml^{-1}$ pronase, 0.5 mg $ml^{-1}$ collagenase and 0.5 mg $ml^{-1}$ DNAase, dissolved in growth medium without serum, followed by neutralisation of the pronase by adding Eagles Minimal Essential medium (MEM) with 10% foetal calf serum (FCS). To remove the enzymes the cells were centrifuged and resuspended in MEM + 10% FCS; any remaining clumps were broken up by syringing through a 19 gauge needle. This procedure has been shown by microscopy to achieve a single cell suspension, with a cell yield of 6.5 ± 0.6 × $10^7$ cells $g^{-1}$ and a mean plating efficiency of 0.26 ± 0.02. The cells were counted with a Coulter counter and appropriate numbers were plated onto 9 cm petri dishes that had been prepared with a feeder layer of 2 × $10^5$ lethally-irradiated V79 379A Chinese hamster cells in MEM + 10% FCS. Each individual tumour was plated at two cell densities on four dishes each, except for unirradiated controls that were plated at one cell density on 6 dishes. There was no consistent difference in cell yield nor in tumour weight in the different treatment groups. All dishes were incubated (under an atmosphere of 5% $O_2$, 5% $CO_2$, balance nitrogen) at 37°C for 10 days, after which the medium was removed and the colonies were then fixed and stained with 0.2% crystal violet in 70% ethanol.

*Enhancement ratios*

Iso-effective doses (± 95% CL), obtained from the logit fits to the local control probability data, were used to calculate enhancement ratios (ERs) as the ratio of the X-ray dose in air to the dose with the sensitiser combination at the same level of damage. Dose response curves drawn through the survival data (Figure 3) were obtained by fitting a third-order polynomial equation to the logarithmically transformed surviving fraction (SF)‡ of each individual tumour against dose, using non-linear least-squares regression. The parameters obtained from the fits to each survival dose-response curve were then used to calculate iso-effective doses (± 95% CL), and from these, enhancement ratios at three different levels of survival were obtained in the manner described above. The significance of differences in the dose-modifying factors between dose-response curves, for the different treatment groups, was estimated by carrying out a *t*-test on pairs of enhancement ratios and their respective errors.

### Results

Plasma clearance after a single i.p. injection of 0.1–0.5 mg $g^{-1}$ of nicotinamide in CBA mice is shown in Figure 1. There was rapid absorption of the compound and peak plasma levels occurred for all dose groups within the first 10–15 min. The levels achieved were dose dependent, with a nearly five-fold lower peak concentration for 0.1 mg $g^{-1}$ compared with 0.5 mg $g^{-1}$. For all dose levels the clearance was linear in the first 3–6 h. The fits to the data were calculated, over the time intervals indicated in Figure 1, using non-linear least squares regression; the apparent half-lives are shown in Table I along with other pharmacokinetic information. There was a trend for an increase in $t_{\frac{1}{2}}$ as the

*(Pantak; Astrophysics Research Ltd. Vale Rd, Windsor, UK)

$$^{\ddagger}SF = \frac{\text{Plating efficiency of treated tumours}}{\text{Mean plating efficiency of control tumours}}$$

**Figure 1** Plasma clearance in non-tumour bearing CBA male mice as a function of time (two mice per time point) after a single i.p. injection of 0.1 to 0.5 mg $g^{-1}$ of nicotinamide. The lines represent non-linear least square regression fits using the initial 0 to 3 time points for 0.1 mg $g^{-1}$ (■), 0 to 5 h for 0.2 mg $g^{-1}$ (◆) and 0.3 mg $g^{-1}$ (▼), and 0 to 6 h for 0.5 mg $g^{-1}$ (●).

dose of nicotinamide was increased but the difference was significant only when comparing 0.1 mg $g^{-1}$ with the other doses ($P<0.01$).

Figure 2 shows dose-response curves from two separate local tumour control experiments, in mice breathing air alone, carbogen alone, or carbogen with 0.1–0.5 mg $g^{-1}$ nicotinamide injected 1 h before each fraction. The responses in all repeat schedules were remarkably similar, and there was no significant difference in the isoeffective doses obtained when the fits were done separately for each experiment (data not shown). Therefore, the fits shown in Figure 2 were obtained by pooling the data. These data show a significant increase in tumour radiosensitisation for all four doses of nicotinamide combined with carbogen compared with tumours treated under air-breathing conditions ($P<0.00001$). Carbogen alone was also very effective and, as shown in **d**, a substantial part of the sensitising effect observed with CON was achieved by carbogen.

Cell survival curves for tumours irradiated in air, carbogen alone or carbogen combined with nicotinamide doses from 0.05 to 0.5 mg $g^{-1}$ are shown in Figure 3. Relative to air, radiosensitisation was seen with all CON treatments but the magnitude of the effect was radiation dose dependent; below 0.5 Gy per fraction sensitisation was not observed. Nicotinamide doses of 0.05 to 0.3 mg $g^{-1}$ all gave similar enhancements of tumour damage and these were significantly lower than those observed with the two highest doses tested. Carbogen alone gave a small but significant increase in radiosensitisation.

Tables II and III summarise the enhancement ratios for the different combinations investigated with both assays compared with treatments under air-breathing conditions. Relative to air, all ERs were significantly different (Tables IV and V). There was a trend towards a progressive increase in radiosensitisation as the dose of nictotinamide was increased. Radiosensitisation with carbogen alone was significantly lower in the excision assay than in the local control assay. Our earlier experience with normobaric oxygen and carbogen has been that larger ERs are generally seen with local tumour control than with other assays in which less cell kill is induced (Rojas *et al.*, 1990*a*). With CON, radiosensitisation was non-significantly less with the excision assay than that observed with local control probability (Table II *cf* Table III). With the former assay, ERs increased with increasing level of cell kill for all 6 different sensitiser combinations. Above $5 \times 10^{-1}$ surviving fraction no sensitising effect was observed. At the lower levels of cell survival radiosensitisation with this assay was similar to that seen with local tumour control. With this assay, ERs at the $TCD_{50}$ level (dose required to control 50% of tumours) were similar to those calculated at other levels of effect (data not shown).

### Discussion

In experimental radiotherapy, tumour radiosensitisation with carbogen combined with nicotinamide differs from that seen with other oxygen-mimetic radiosensitisers in that the benefit does not decrease with fractionation. At a clinically relevant radiation dose per fraction of 2 Gy, enhancement ratios of 1.9 and 2.1 have been observed with carbogen plus 0.5 mg $g^{-1}$ of nicotinamide (Rojas, 1992). Since a large and significant therapeutic gain has been seen in mice (Haustermans, 1992; Kjellen *et al.*, 1991; Rojas *et al.*, unpublished), CON could be a very effective and non-toxic sensitiser for use in humans. In clinical radiotherapy however, the effectiveness of the carbogen-nicotinamide combination will be limited by the dose of nicotinamide that can be administered to patients, by the ability of carbogen to increase tumour $pO_2$, and by the extent of normal tissue sensitisation.

Until now, all the fractionated radiation studies with CON in rodents have been carried out with nicotinamide doses of 0.5 mg $g^{-1}$, which are above those that can be realistically achieved in man. The vitamin has been used extensively in human dermatological and psychiatric disorders, and more recently, in the prevention and treatment of diabetes type I (Green, 1970; Hawkins, 1968; Hoffer, 1971; Vague *et al.*, 1989). Doses of up to 12 g per day (~0.17 mg $g^{-1}$ for a 70 kg man) have been administered over many months. Few side-effects, all of which are reversible, have been reported in the literature (Hoffer, 1971; Winter & Boyer, 1973; Zackheim *et al.*, 1981). Two surveys conducted in the USA reported that the major limiting toxicity with the vitamin was liver failure, with an incidence of one in 2000–3000 patients (Zackheim *et al.*, 1981). Therefore, from a drug toxicity point of view, 6–9 g (~0.09–0.13 mg $g^{-1}$ for a 70 kg man) per day for the duration of an accelerated or a conventional radiotherapy regime should be feasible.

Our studies show that nicotinamide doses of 0.05 to 0.5 mg $g^{-1}$, when combined with carbogen, significantly increase the radiosensitivity of rodent CaNT tumours compared with fractionated X-ray treatments under air-breathing conditions (Figures 2 and 3). Enhancement ratios obtained with CON (0.05–0.5 mg $g^{-1}$) were significantly larger than those seen with carbogen alone (Tables IV and V). In the local control assay, although the ER for CON (0.1 mg $g^{-1}$)

**Table I** Plasma pharmacokinetics of varying nicotinamide (NAM) doses

| *Species* | *Dose* | *Time of [Peak] min* | *[Peak] μmol $l^{-1}$* | *[NAM] at 1 h μmol $ml^{-1}$* | *$t_{½}$ h (± 95% CL)* | *Reference* |
|---|---|---|---|---|---|---|
| *Mice* | 0.1 mg $g^{-1}$ | 10 | 1.04 | 0.69 | 1.44 ± 0.16 | These data |
| | 0.2 mg $g^{-1}$ | 10 | 1.81 | 1.23 | 1.95 ± 0.18 | |
| | 0.3 mg $g^{-1}$ | 10 | 2.89 | 1.98 | 1.95 ± 0.18 | |
| | 0.5 mg $g^{-1}$ | 15 | 4.85 | 3.67 | 2.19 ± 0.15 | |
| *Man*[a] | 6 g | 44–180 | 0.7–1.1 | | 8.7–9.2 | Stratford *et al.*, 1992 |
| | 6 g | 5–49 | 1.0–1.6 | | 6.0–11.5 | Horsman *et al.*, 1993 |

[a]Range observed in four human volunteers.

**Figure 2** Local control probability for CaNT tumours treated with 10 fractions in 5 days in air (**a**: △, ▲), with carbogen plus 0.1 mg $g^{-1}$ (**a**: □, ■), 0.2 mg $g^{-1}$ (**b**: ◆), 0.3 mg $g^{-1}$ (**c**: ▽, ▼), 0.5 mg $g^{-1}$ of nicotinamide (**d**: ○, ●), or carbogen alone (**d**: ■). The dashed line (**b** to **d**) represents the fit to the data for irradiations in air. Errors are 95% CL. The lines through the data were obtained by fitting pooled data from two separate experiments (open and closed symbols).

**Figure 3** Cell survival of CaNT tumour clonogens (each point represents a mean of six mice per dose group ± 1 s.e.m.) with an *in vivo/in vitro* excision assay. Tumours were irradiated with a 10 fractions in 5 days schedule, in air (○), carbogen alone (△) or carbogen combined with the indicated doses of nicotinamide (●).

was greater than that for carbogen alone, it failed to reach significance. Carbogen alone is an effective radiosensitiser but its efficacy varies from one experiment to another; an ER as low as 1.2 was seen in CaNT tumours using the same fractionated regime (Kjellen *et al.*, 1991; Rojas, 1992). By contrast, the combination of carbogen with nicotinamide gives large and reproducible enhancements of tumour damage (Rojas, 1992; these data). In mice, there is no evidence of treatment associated toxicity with CON either in conventional schedules with 30 fractions in 6 weeks or in accelerated regimes with 20–40 fractions in 3–4 weeks (Rojas *et al.*, unpublished).

In the present study nicotinamide plasma concentration in CBA mice was linearly related to administered dose on a mg $g^{-1}$ basis; a similar linear relationship has also been observed in CDF1 mice and humans (Horsman, 1992; Horsman *et al.*, 1993; Stratford *et al.*, 1992). Figure 4 shows peak plasma levels in man compared with peak levels and levels at 1 h in rodents. A single oral dose of either 6 or 9 g of nicotinamide in man achieved a peak concentration comparable with peak levels obtained in mice after an i.p. administration of 0.1 or 0.2 mg $g^{-1}$, respectively (Horsman *et al.*, 1993; Stratford, *et al.*, 1992; Stratford *et al.*, unpublished). With the local control assay, a decrease of about 5% in the efficacy of CON was seen when the dose of nicotinamide was reduced from 0.5 to 0.3 and 0.2 mg $g^{-1}$, and a further 10% reduction when reduced to 0.1 mg $g^{-1}$. Although there was almost a three-fold difference in plasma concentration between 0.5 mg $g^{-1}$ and 0.2 mg $g^{-1}$ (Figure 4, top), only a small further gain in local control is achieved with CON by increasing the dose of nicotinamide above 0.2 mg $g^{-1}$. As shown in Figure 4 (bottom), the change in radiosensitisation *in vivo* over a range of nicotinamide plasma levels is best fitted by an exponential rather than by a linear relationship (the exponential fit gives a lower residual sum of errors). Others have previously reported a similar finding for nicotinamide alone but at a higher drug-dose level (Horsman *et al.*, 1989). Relative to air breathing mice, significant sensitisation is seen with CON at nicotinamide plasma concentrations of 0.7–1.6 μmol $ml^{-1}$ that are achieved in humans (Table I *cf.* Figure 4). Peak plasma levels seen in man indicate that 6 and 9 g doses, if combined with carbogen, could achieve ERs higher than those observed in mice with 0.1–0.2 mg $g^{-1}$ of nicotinamide (Table I; Stratford *et al.*, unpublished).

So far all the fractionated studies in rodent tumours have been performed with a 1 h gap between nicotinamide admini-

**Table II** Enhancement ratios for carbogen alone or with varying nicotinamide (NAM) doses relative to air-breathing mice, using a local tumour control assay

| *NAM* ($mg\,g^{-1}$) | *Gas* | *Dose/fraction* (*Gy*) | $TCD_{50}$ (*Gy*) (± 95% CL) | *ER* (± 95% CL) |
|---|---|---|---|---|
| 0 | Air | 5.5–8.6 | 75.28 ± 2.34 | |
| 0 | Carbogen | 3.7–6.8 | 50.78 ± 4.75 | 1.48 ± 0.15 |
| 0.1 | Carbogen | 3.2–5.5 | 48.43 ± 3.37 | 1.55 ± 0.12 |
| 0.2 | Carbogen | 3.8–5.5 | 43.93 ± 2.91 | 1.71 ± 0.13 |
| 0.3 | Carbogen | 3.1–5.2 | 42.75 ± 2.57 | 1.76 ± 0.12 |
| 0.5 | Carbogen | 3.0–5.1 | 40.65 ± 1.88 | 1.85 ± 0.10 |

**Table III** Enhancement ratios (± 95% CL) for carbogen alone or with varying nicotinamide (NAM) doses relative to air-breathing mice, using an excision assay

| *NAM* ($mg\,g^{-1}$) | *Dose/fraction* (*Gy*) | *ER* @ (*SF* = 0.10) | *ER* @ (*SR* = 0.02) | *ER* @ (*SF* = 0.01) | *ER* @ (*SF* = 0.003) |
|---|---|---|---|---|---|
| 0 | 0.4–2.5 | 1.11 ± 0.12 | 1.19 ± 0.10 | | |
| 0.05 | 0.3–2.0 | 1.22 ± 0.12 | 1.31 ± 0.10 | | |
| 0.1 | 0.3–2.0 | 1.26 ± 0.14 | 1.41 ± 0.12 | 1.46 ± 0.12 | |
| 0.2 | 0.3–2.0 | 1.42 ± 0.16 | 1.48 ± 0.13 | 1.50 ± 0.13 | |
| 0.3 | 0.3–2.0 | 1.29 ± 0.13 | 1.39 ± 0.11 | 1.43 ± 0.11 | |
| 0.4 | 0.3–2.0 | 1.51 ± 0.23 | 1.69 ± 0.18 | 1.75 ± 0.17 | 1.85 ± 0.18 |
| 0.5 | 0.3–2.0 | 1.40 ± 0.16 | 1.63 ± 0.14 | 1.70 ± 0.14 | 1.82 ± 0.15 |

**Table IV** Probability that pairs of ERs are not significantly different from each other. Local tumour control assay; *P* values calculated at the $TCD_{50}$ level

| | *Carbogen + Nam 0* | *Carbogen + Nam 0.1* | *Carbogen + Nam 0.2* | *Carbogen + Nam 0.3* | *Carbogen + Nam 0.5* |
|---|---|---|---|---|---|
| *P* relative to air | <0.00001 | <0.00001 | <0.00001 | <0.00001 | <0.00001 |
| *P* relative to Carbogen + Nam 0 $mg\,g^{-1}$ | | 0.2[a] | 0.01 | 0.003 | 0.0001 |
| *P* relative to Carbogen + Nam 0.1 $mg\,g^{-1}$ | | | 0.04 | 0.008 | 0.0001 |
| *P* relative to Carbogen + Nam 0.2 $mg\,g^{-1}$ | | | | 0.3[a] | 0.05 |
| *P* relative to Carbogen + Nam 0.3 $mg\,g^{-1}$ | | | | | 0.1[a] |

Statistical significance of the differences in ERs obtained with carbogen combined with the indicated doses of nicotinamide. Comparisons were made between pairs of ERs for all combinations of treatment schedules.[a]Not significant at 0.05 level.

**Table V** Probability that pairs of ERs are not significantly different from each other. Excision assay: *P* values at SF = 0.02

| | *Carbogen + Nam 0* | *Carbogen + Nam 0.05* | *Carbogen + Nam 0.1* | *Carbogen + Nam 0.2* | *Carbogen + Nam 0.3* | *Carbogen + Nam 0.4* | *Carbogen + Nam 0.5* |
|---|---|---|---|---|---|---|---|
| *P* relative to air | 0.007 | 0.0004 | <0.00001 | <0.00001 | <0.00001 | <0.00001 | <0.00001 |
| *P* relative to Carbogen + Nam 0 $mg\,g^{-1}$ | | 0.05 | 0.004 | 0.0005 | <0.005 | <0.00001 | <0.00001 |
| *P* relative to Carbogen + Nam 0.05 $mg\,g^{-1}$ | .. | | 0.1[a] | 0.02 | 0.2[a] | 0.0003 | 0.0003 |
| *P* relative to Carbogen + Nam 0.1 $mg\,g^{-1}$ | | | | 0.2[a] | 0.6[a] | 0.007 | 0.01 |
| *P* relative to Carbogen + nam 0.2 $mg\,g^{-1}$ | | | | | 0.9[a] | 0.03 | 0.06 |
| *P* relative to Carbogen + Nam 0.3 $mg\,g^{-1}$ | | | | | | 0.004 | 0.005 |
| *P* relative to Carbogen + Nam 0.4 $mg\,g^{-1}$ | | | | | | | 0.7[a] |

Statistical significance of the differences in ERs obtained with carbogen combined with the indicated doses of nicotinamide. Comparisons were made between pairs of ERs for all combinations of treatment schedules.[a]Not significant at 0.05 level.

**Figure 4** **a**, peak plasma concentrations (■) and plasma concentrations at 1 h (□) after a single i.p. dose of 0.1 to 0.5 mg g$^{-1}$ of nicotinamide to CBA non-tumour bearing mice. For comparison peak plasma levels seen in four human volunteers with escalating oral doses of 1, 2, 4 (O), 6 (Δ) and 9 g (◇) of the vitamin are shown (O, Δ: Stratford *et al.*, 1992; ◇: Stratford *et al.*, unpublished). Lines are least squares fits. **b**, Enhancement ratios (± 95% CL) at the $TCD_{50}$ level for carbogen alone or combined with varying doses of nicotinamide, plotted against plasma concentration at one hour after injection of 0, 0.1, 0.2, 0.3 and 0.5 mg g$^{-1}$ nicotinamide (▲, ■, ◆, ▼, ●, respectively). Included are data from Kjellen, *et al.*, 1991 (Δ, O). Lines are linear (dot-dashed) and exponential (dashed) fits to all the data.

stration and irradiation, which is about 30 min after peak concentrations are observed in CaNT tumours (Stratford & Dennis, in preparation). Previously, Horsman *et al.* (1989) showed tumour sensitisation decreased with decreasing dose of nicotinamide, and only a 10% increase in radiosensitivity could be obtained with 0.1–0.2 mg g$^{-1}$. However, by reducing the interval between injection and irradiation to 20 min, (i.e. the time at which peak tumour levels were seen for all dose levels), they showed that ERs with 0.1–0.2 mg g$^{-1}$ were comparable to those obtained with 0.4–1.0 mg g$^{-1}$. This suggests that concentration at the time of irradiation is the factor that mainly determines the degree of tumour radiosensitisation (Horsman *et al.*, 1993). However, if length of exposure to nicotinamide plays any role in tumour radiosensitisation, a dose of 6 g in man could be more effective than 0.1 mg g$^{-1}$ in the mouse, since the half-life of the vitamin in man is 3–8 times longer than that in rodents (Table I). Enhancement ratios *in vivo* with CON reported here and at lower doses per fraction (Rojas, 1992) are relatively high if explained solely on the basis of complete sensitisation of hypoxic cells, and other mechanisms (e.g. selective repair inhibition of tumour cells) have not yet been excluded. Although no evidence of repair inhibition was seen in rodent gut, skin and kidney (Horsman *et al.*, 1987; Rojas *et al.*, 1990*b*; Joiner *et al.*, unpublished), nicotinamide can change the slopes of tumour cell survival curves, suggesting that the radiosensitisation observed was not only due to a reduction in the fraction of hypoxic cells (Horsman *et al.*, 1987).

Microelectrode measurements show that carbogen is very effective at increasing $pO_2$ in human tumours within 2–10 min of breathing the gas (Falk *et al.*, 1992; Martin *et al.*, 1993), and no toxic side-effects are seen in patients even when administered for periods of up to 2 h each day (Dische *et al.*, 1992; Rubin *et al.*, 1979). Although in a large series of patients treated with carbogen and radiotherapy there was no evidence of increased early or late morbidity (Rubin *et al.*, 1979), in a small, more recent, series of patients one case of radiation myelitis and one of skin necrosis did occur (Dische *et al.*, 1992); and with hyperbaric oxygen increased morbidity was observed in cartilage, bowel and spinal cord (Dische, 1983). A palliative, pilot study of 6 fractions in 21 days in Ca breast has shown no evidence of increased acute normal tissue reactions in patients treated with a 6 g dose of nicotinamide given 1.5 h before each fraction plus carbogen given 4–10 min before and during treatment (Saunders & Dische, 1991, pers. comm). However, some degree of normal tissue sensitisation should be anticipated with CON in clinical radiotherapy, and spinal cord and cartilage are of particular concern.

There is considerable interest in the clinical application of carbogen and nicotinamide in both palliative and curative radiotherapy. Animal data now show that very effective radiosensitisation in clinically-relevant experimental regimes can be obtained by combining these methods of attacking tumour hypoxia, and mouse-man comparisons suggest that CON could be a most effective and non-toxic radiosensitiser for use in clinical radiotherapy. However, more laboratory work must be done to assess further its therapeutic potential, to identify tumour parameters that can be used as prognostic indicators and to identify the intrinsic mechanism(s) of action of nicotinamide.

We wish to thank Dr M.C. Joiner for useful and critical discussions, Mrs J. Collier, Miss E. Kelleher, Mrs A. Calvert, Mrs B. Kempton and Mr P. Russell for technical assistance. This project was entirely funded by the Cancer Research Campaign.

### References

CHAPLIN, D.J., HORSMAN, M.R. & AOKI, D.S. (1991). Nicotinamide, Fluosol DA and carbogen: a strategy to reoxygenate acutely and chronically hypoxic cells *in vivo*. *Br. J. Cancer*, **63**, 109–113.

DISCHE, S. (1983). The clinical use of hyperbaric oxygen and chemical hypoxic cell sensitizers. In *The Biological Basis of Radiotherapy*, Steel, G.G., Adams, G.E. & Peckham, M.J. (eds) pp. 225–237. Elsevier, Amsterdam.

DISCHE, S., ROJAS, A., RUGG, T., HONG, A. & MICHAEL, B.D. (1992). Carbogen breathing: a system for use in man. *Br. J. Radiol.*, **65**, 87–90.

FALK, S.J., WARD, R. & BLEEHEN, N.M. (1992). The influence of carbogen breathing on tumour tissue oxygenation in man evaluated by computerised $pO_2$ histography. *Br. J. Cancer*, **66**, 919–924.

FOWLER, J.F. & DENEKAMP, J. (1979). A review of hypoxic cell radiosensitization in experimental tumors. *Pharmac. Therap.*, **7**, 413–444.

GREEN, R.G. (1970). Subclinical pellagra: its diagnosis and treatment. *Schizophrenia*, **2**, 70–79.

HAUSTERMANS, K., LANDUYT, W., VANACKER, B., VAN DER KOGEL, A.J. & VAN DER SCHUEREN, E. (1992). Influence of combined use of nicotinamide and carbogen on spinal cord tolerance. *Radiother. Oncol.*, **24 (suppl)**, 114.

HAWKINS, D.R. (1968). Treatment of schizophrenia based on the medical model. *J. Schizophrenia*, **2**, 3–10.

HENDRY, J.H. (1979). Quantitation of the radiotherapeutic importance of naturally-hypoxic normal tissues from collated experiments with rodents using single doses. *Int. J. Radiat. Oncol. Biol. Phys.*, **5**, 971–976.

HILL, R.P. (1986). Sensitizers and radiation dose fractionation: results and interpretations. *Int. J. Radiat. Oncol. Biol. Phys.*, **12**, 1049–1054.

HOFFER, A. (1971). Megavitamin $B_3$ therapy for schizophrenia. *Canad. Psychiat. Ass. J.*, **16**, 499–504.

HORSMAN, M.R., CHAPLIN, D.J. & BROWN, J.M. (1987). Radiosensitization by nicotinamide *in vivo*: a greater enhancement of tumor damage compared to that of normal tissues. *Radiat. Res.*, **109**, 479–489.

HORSMAN, M.R., CHAPLIN, D.J. & BROWN, J.M. (1989). Tumor radiosensitization by nicotinamide: a result of improved perfusion and oxygenation. *Radiat. Res.*, **118**, 139–150.

HORSMAN, M.R. (1992). Carbogen and nicotinamide: expectations too high? *Radiother. Oncol.*, **24**, 121–122.

HORSMAN, M.R., HØYER, M., HONESS, D., DENNIS, I. & OVERGAARD, J. (1993). Nicotinamide pharmacokinetics in humans and mice: a comparative assessment and the implications for radiotherapy. *Radiother. Oncol.*, **27**, 131–139.

KJELLEN, E., JOINER, M.C., COLLIER, J.M., JOHNS, H. & ROJAS, A. (1991). A therapeutic benefit from combining normobaric carbogen or oxygen with nicotinamide in fractionated X-ray treatments. *Radiother. Oncol.*, **22**, 81–91.

MARTIN, L., LARTIGAU, E., WEEGER, P., LAMBIN, P., LERIDANT, A.M., LUSINCHI, A., WIBAULT, P., ESCHWEGE, F., LUBOINSKI, B. & GUICHARD, M. (1993). Changes in the oxygenation of head and neck tumours during carbogen breathing. *Radiother. Oncol.*, **27**, 123–130.

OVERGAARD, J., SAND HANSEN, H., LINDELOV, B., OVERGAARD, M., JORGENSEN, K. & RASMUSSON, B. (1991). Nimorazole as a hypoxic radiosensitizer in the treatment of supraglottic larynx and pharynx carcinoma. First report from the Danish head and neck cancer study (Dahanca). *Radiother. Oncol.*, **20**, 143–149.

OVERGAARD, J. (1992). Importance of tissue hypoxia in radiotherapy. A meta-analysis of controlled clinical trials. *Radiother. Oncol.*, **24 (suppl)**, 64.

ROJAS, A., CARL, U. & REGHEBI, K. (1990*a*). Effect of normobaric oxygen on tumor radiosensitivity: fractionated studies. *Int. J. Radiat. Oncol. Biol. Phys.*, **18**, 547–553.

ROJAS, A., KJELLEN, E. & JOINER, M.C. (1990*b*). Does nicotinamide inhibit repair of radiation damage *in vivo*? *Br. J. Radiol.*, **63**, 914.

ROJAS, A. (1992). ARCON: accelerated radiotherapy with carbogen and nicotinamide. *Br. J. Radiol.*, **24**, 174–178.

RUBIN, P., HANLEY, J., KEYS, H.M., MARCIAL, V. & BRADY, L. (1979). Carbogen breathing during radiation therapy. *Int. J. Radiat. Oncol. Biol. Phys.*, **5**, 1963–1970.

STEWART, F.A., DENEKAMP, J. & RANDHAWA, V.S. (1982). Skin sensitization by misonidazole: a demonstration of uniform mild hypoxia. *Br. J. Cancer*, **45**, 869–877.

STRATFORD, M.R.L. & DENNIS, M.F. (1992). High-performance liquid chromatographic determination of nicotinamide and its metabolites in human and murine plasma and urine. *J. Chromatog.*, **582**, 145–151.

STRATFORD, M.R.L., ROJAS, A., HALL, D.W., DENNIS, M.F., DISCHE, S., JOINER, M.C. & HODGKISS, R.J. (1992). Pharmacokinetics of nicotinamide and its effects on blood pressure, pulse, and body temperature in normal human volunteers. *Radiother. Oncol.*, **25**, 37–42.

VAGUE, P., PICQ, R., BERNAL, M., LASSMAN-VAGUE, V. & VIALETTES, B. (1989). Effect of nicotinamide treatment on the residual insulin secretion in type 1 (insulin-dependent) diabetic patients. *Diabetol.*, **32**, 316–321.

WINTER, S.L. & BOYER, J.L. (1973). Hepatic toxicity from large doses of vitamin $B_3$ (nicotinamide). *New England J. Med.*, **289**, 1180–1182.

ZACKHEIM, H.S., VASILY, D.B., WESTPHAL, M.L. & HASTINGS, C.W. (1981). Reactions to niacinamide. *J. Amer. Ac. Derm.*, **4**, 736–737.